# Coding and noncoding somatic mutations in candidate genes in basal cell carcinoma

**DOI:** 10.1038/s41598-020-65057-2

**Published:** 2020-05-14

**Authors:** Maria Giovanna Maturo, Sivaramakrishna Rachakonda, Barbara Heidenreich, Cristina Pellegrini, Nalini Srinivas, Celia Requena, Carlos Serra-Guillen, Beatriz Llombart, Onofre Sanmartin, Carlos Guillen, Lucia Di Nardo, Ketty Peris, Maria Concetta Fargnoli, Eduardo Nagore, Rajiv Kumar

**Affiliations:** 1 Department of Dermatology, Department of Biotechnology and Applied Clinical Sciences, University of L’Aquila, L’Aquila, Italy; 2 Division of Functional Genome Analysis, German Cancer Research Center, Heidelberg, Germany; 3 Division of Molecular Genetic epidemiology, German Cancer Research Center, Heidelberg, Germany; 4 Department of Dermatology, Instituto Valenciano de Oncología, València, Spain; 5 Institute of Dermatology, Catholic University of Sacred Heart, Fondazione Policlinico Universitario A. Gemelli, IRCCS, Rome, Italy

**Keywords:** Cancer epigenetics, Predictive markers

## Abstract

Basal cell carcinoma (BCC) represents the most commonly diagnosed human cancer among persons of European ancestry with etiology mainly attributed to sun-exposure. In this study we investigated mutations in coding and flanking regions of *PTCH1* and *TP53* and noncoding alterations in the *TERT* and *DPH3* promoters in 191 BCC tumors. In addition, we measured CpG methylation within the *TERT* hypermethylated oncological region (THOR) and transcription levels of the reverse transcriptase subunit. We observed mutations in *PTCH1* in 58.6% and *TP53* in 31.4% of the tumors. Noncoding mutations in *TERT* and *DPH3* promoters were detected in 59.2% and 38.2% of the tumors, respectively. We observed a statistically significant co-occurrence of mutations at the four investigated loci. While *PTCH1* mutations tended to associate with decreased patient age at diagnosis; *TP53* mutations were associated with light skin color and increased number of nevi; *TERT* and *DPH3* promoter with history of cutaneous neoplasms in BCC patients. Increased reverse transcriptase subunit expression was observed in tumors with *TERT* promoter mutations and not with THOR methylation. Our study signifies, in addition to the protein altering mutations in the *PTCH1* and *TP53* genes, the importance of noncoding mutations in BCC, particularly functional alterations in the *TERT* promoter.

## Introduction

Basal cell carcinoma (BCC) accounts for about 80 percent of all skin cancers and it is the most commonly diagnosed neoplasm among the Caucasian population^1–3^. The tumor originates from stem cells within hair follicles or the interfollicular epidermis and infundibulum undergoing ultraviolet (UV) radiation induced mutagenesis^4–6^. Basal cell carcinoma rarely metastasizes; however, due to sheer number of people affected, the disease poses a considerable health hazard as it causes extensive morbidity through local invasion and tissue destruction^7^. The most common genetic alterations in BCC involve the Hedgehog receptor patched 1 (*PTCH1*) and the cell cycle regulator *TP53* and the mutations in both genes predominantly reflect UV etiology^8^. Exome sequencing based studies, besides denoting BCC as the cancer with highest mutation burden, have also identified less frequent alterations in genes other than *PTCH1* and *TP53*^8,9^.

Germline mutations *PTCH1* also occur in patients with Gorlin syndrome, a Mendelian disease with a high prevalence of BCC^10^. Aberrant activation of the Hedgehog pathway is common in sporadic BCC either through mutations in the *PTCH1* gene, activating mutations in smoothened (*SMO*) or loss-of-function mutations in suppressor of fused homolog (*SUFU*)^9,11,12^. Ubiquitous hyper-activation of the Hedgehog has led to development of inhibitors targeting G-protein coupled receptor Smoothened for treatment of advanced BCC^11,12^. While, the studies based on exome sequencing have confirmed the centrality of *PTCH1* mutations in BCC, the variants within smoothened are reportedly causal in resistance to the inhibitors^8^.

*TP53* encodes a transcription factor, which is a tumor suppressor involved in cellular stress responses including DNA damage, oxidative stress, oncogenic hyperproliferation, pathogenic stimuli, UV-induced pigmentation^13,14^. Due to the criticality of p53 mediated transcription in tumor suppression, mutations in *TP53* mainly occur within the DNA binding domain that cluster at several hotspot amino-acid residues^15,16^. A large proportion of *TP53* mutations in skin cancers including BCC are typically C > T and CC > TT transitions at dipyrimidines localized within several specific mutational hotspots due to hyper proneness to UV-induced damage^15,17–19^. BCC tumors have been shown to arise in skin containing clones of cells with stabilized and mostly mutant p53 protein^20,21^.

Besides the frequent canonical coding region alterations in *PTCH1* and *TP53*, noncoding mutations within the core promoter of the gene encoding telomerase reverse transcriptase (*TERT*) occur at elevated frequencies in BCC^22–25^. Reported initially in melanoma, the *TERT* promoter mutations are frequent in multiple cancers, with some exceptions, arising from tissues with low rates of self-renewal^22,26–28^. Those promoter mutations create *de novo* sites where binding of ETS transcription factors leads to increased transcription through epigenetic histone modification^26,29^. Methylation within the *TERT* hypermethylated oncological region (THOR) of the same promoter also de-represses *TERT* transcription^30^. Noncoding mutations in a bidirectional *DPH3* promoter region with unknown functional consequences are also frequent in different skin cancers including BCC^31^.

In this study, we sequenced 191 BCC lesions and 115 corresponding normal skin surrounding tumors for mutations in the *PTCH1* and *TP53* genes, and the *TERT* and *DPH3* promoters. We also investigated CpG methylation at the *TERT* promoter between −560 to −774 bp region from the ATG start site that falls within the THOR. The effect of the *TERT* promoter mutations and THOR methylation on the transcription of the catalytic subunit of the telomerase was also measured. In addition, we also investigated the correlation between *TERT* promoter mutations and telomere length in BCC tumors.

## Results

### PTCH1 mutations

We detected 137 *PTCH1* mutations in 105 tumors; of those 44 tumors also showed loss of heterozygosity including focal deletions in 4 tumors (Table 1**;** Supplementary Figure 1; Supplementary Table 1 and 2). In addition 7 tumors showed only loss of heterozygosity without a mutation on the remaining allele. One mutation each was detected in 79 (41%) tumors, 21 (11%) tumors carried two mutations each, four tumors had three mutations each and one tumor had four mutations.

**Table 1:** Mutations in *TERT* promoter, *DPH3* promoter, *TP53* and *PTCH1*.

	BCC (total n = 191)	%
***TERT*** **promoter**		
**Number of lesions with mutations**	**113**	**59.2**
**−146C** > **T**	67	35.1
**−124C** > **T**	14	7.3
**Tandem mutations**	16	8.4
−124_125 CC > TT	3	1.6
−138_139CC > TT	11	5.8
−138_139CC > TT, −125C > T	1	0.5
−101_102CC > TT, −124C > T	1	0.5
**Others:**	16	8.4
−101C > T	4	2.1
−101C > T, −126C > T	1	0.5
−125C > T	1	0.5
−126C > T	1	0.5
−146C > T, −101C > T	3	1.6
−146C > T, −126C > T	1	0.5
−146C > T, −149C > T	3	1.6
−146C > T; −148C > T	1	0.5
−149C > T	1	0.5
***DPH3*** **promoter**		
**Mutations**	**73**	**38.2**
**−8 C** > **T**	35	18.3
**−9 C** > **T**	14	7.3
**Tandem mutations**	19	9.4
−8_9 CC > TT	17	8.9
−8_9 CC > TT; −12C > T	1	0.5
−8_9 CC > TT; −13C > T	1	0.5
**Others:**	5	2.6
−8 C > T; −12 C > T	1	0.5
−9C > A	2	1.0
−12C > T	2	1.0
***TP53*** **(exon 5–9)**		
**Mutations**^a^	**60**	**31.4**
Tandem mutations (CC > TT)	16	8.4
***PTCH1***	**112**	**58.6**
**Mutations**^b,c^	105	54.9
**Only loss of heterozygosity**	7	3.7

^a^31 tumors with 2 or more alterations.

^b^62 tumors with 2 or more alterations.

^c^44 tumors also showed loss of heterozygosity.

**Figure 1:**
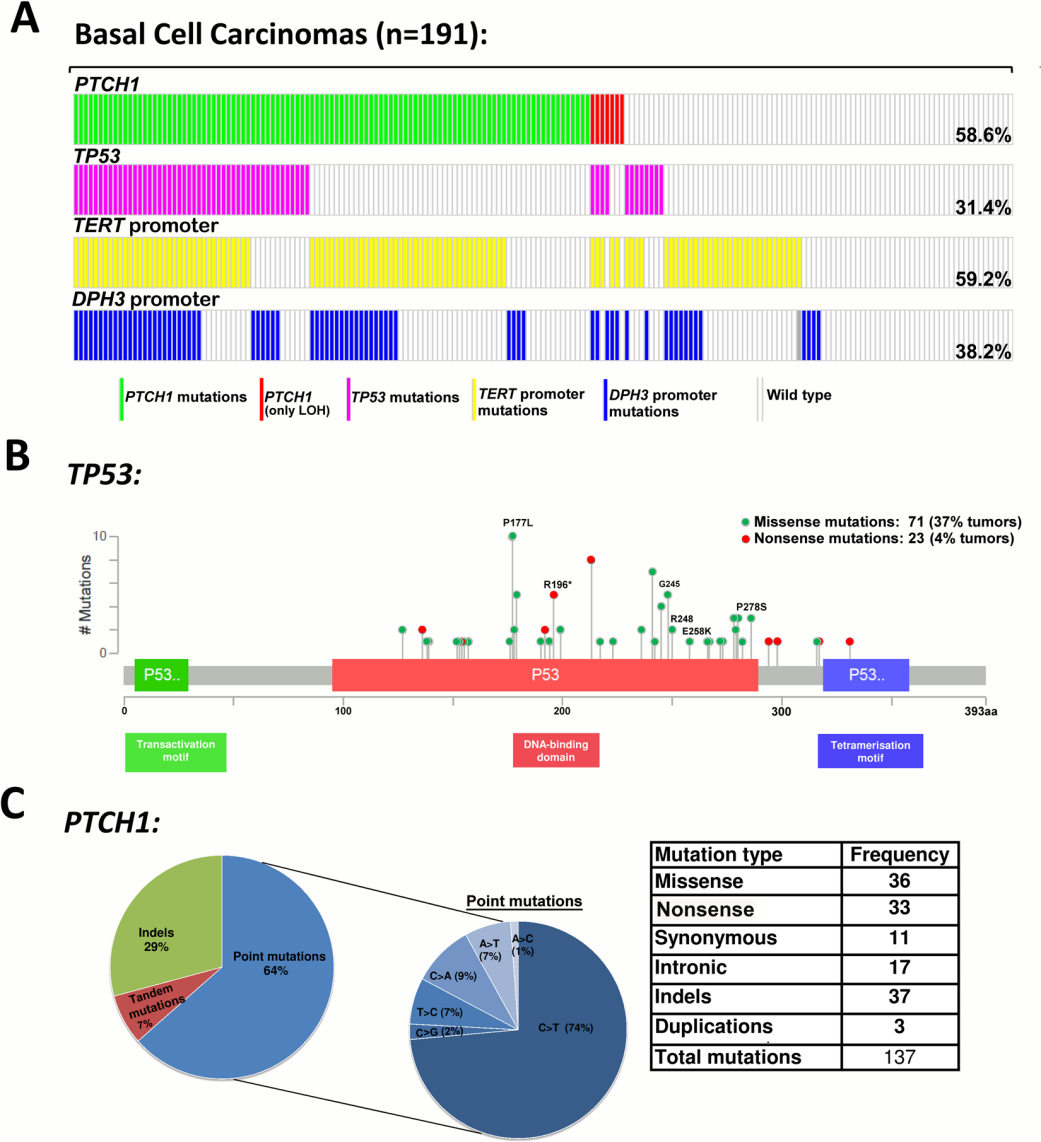
Mutations in skin basal cell carcinoma tumors: (**A**) Distribution of mutation at *TERT* promoter, *DPH3* promoter, *TP53* and *PTCH1* gene. (**B**) Distribution of mutations within different p53 domains. Protein diagram was generated with cBioPortal tools. Hotspot mutations in the gene have been labelled with amino-acid changes. (**C**) Frequency and type of mutations in ***PTCH1*** gene.

The C > T base change was the most frequent mutation with 64 transitions in 56 (29%) tumors, followed by CC > TT tandem mutations, and C > A mutations in 8 tumors each. Thirty-six mutations were missense, 33 were nonsense and 17 mutations were intronic (Fig. 1). We also detected 37 insertion-deletions (indels) that included 25 frame-shift alterations, 6 in-frame deletions, two nonsense, and three intronic. Out of three duplications, two were frame-shift and one in-frame. We also detected 14 mutations at 5′ splice sites and 7 mutations at 3′ splice sites (Supplementary Table 3). Mutations were distributed throughout the gene from exon 3 to exon 23; exon 23 with 12 mutations had the highest number of alterations followed by exons 18 and 6 with 10 mutations each. Most of the missense mutations were in exon 23 while exon 18 had the largest number of nonsense mutations and indels were most frequent in exon 20.

### TP53 mutations

Sixty of the 191 (31%) BCC carried alterations in *TP53*, with 31 (16%) tumors carrying more than one mutation; 24 tumors carried two mutations and 4 tumors carried three mutations and 3 tumors carried four mutations. In total, 100 alterations in the *TP53* gene were identified. The most frequent mutations were C > T transitions with 66 transitions in 48 (25%) tumors, followed by 17 CC > TT tandem transitions in 16 (8%) tumors (Table 1; Supplementary Table 1**;** Supplementary Table 4).

The majority of *TP53* mutations were missense, 71 mutations in 51 tumors (37%) and 23 nonsense mutations were present in 20 tumors (4%). The mutations were distributed within the DNA binding domain of *TP53* (Fig. 1). Hotspot mutations were detected at p.P177 in 8 (4.2%) tumors, at p.R196 in 5 (2.6%) tumors, p.G245 in 5 (2.6%) tumors, p.R248 in 4 (2.1%) tumors, p.E258 in 2 (1%) tumors, and p.P278 in 3 (1.6%) tumors. Other frequent mutation included p.H179Y (c.535 C > T) mutation in exon 5 in 4 (2.1%) tumors. The truncating mutations detected with the highest frequency were the p.R213* in exon 6 (c.637 C > T) identified in 8 (4.2%) tumors and p.R196* in exon 6 (c.586 C > T) in 4 (2.1%) tumors.

### TERT promoter mutations

*TERT* promoter mutations were present in 113 of 191 (59.2%; Fig. 1) lesions with the −146C > T mutation in 67 (35.1%) tumors followed by −124C > T in 14 (7.3%) tumors (Table 1**;** Supplementary Table 1). CC > TT tandem mutations at −138_139, −124_125 and −101_102 bp positions were present in 16 (8.4%) tumors. Eight BCC tumors in addition to the −146C > T hotspot mutation also carried additional alterations and in five tumors alterations other than the recurrent hotspot mutations were detected (Table 1).

### TERT promoter methylation

Pairwise alignment of bisulfite and target genomic sequence using QUMA showed> 85% of homology in 157 (86%) of 183 tumors. Of those 157 tumors, 93 (59%) carried *TERT* promoter mutations while the remaining 64 (41%) were wild type. The methylation at the screened 14 CpG sites was statistically significantly higher in tumors without the *TERT* promoter mutations (99%; 870 of 882 methylation sites) than in the tumors with those mutations (96%; 1219 of 1267 methylation sites; Fisher’s exact P: 0.0006, Mann-Whitney U-test P: 0.003). The standard errors (SE 0.65 vs 0.78%) between the two groups were not statistically significantly different (F test P: 0.12). Comparison of methylation at individual CpG sites showed a statistically significant differences at positions chr5:1,295,731, hg 19 (97% in tumors without mutations vs 80% in tumors with mutations; Fisher’s exact P: 0.003) and chr5:1,295,759 (98% in tumors without mutations vs 90% in tumors with mutations; Fisher’s exact P: 0.05).

Analysis of bisulfite converted DNA after cloning also showed statistically significantly higher methylation in tumors without mutations than in those with mutations (Fisher’s exact P: < 0.00001, Mann-Whitney U-test P: < 0.00001; Supplementary Fig. 2). Pairwise sequence alignment of cloned bisulfite sequence and the target genomic sequence using QUMA showed more than 95% homology for each clone. Individually at the each CpG sites, tumors without the *TERT* promoter mutations had statistically significantly higher methylation than the tumors with mutations. The methylation across all 14 CpG sites ranged between 85% − 98% in tumors without mutations compared to 44% − 91% in tumors with mutations.

### DPH3 promoter mutations

Mutations in the *DPH3* promoter were present in 73 of 191 (38.2%; Fig. 1) tumors. In addition to the frequent C > T transitions observed in 35 (18.3%) tumors at −8 bp and in 14 (7.3%) tumors at −9 bp, CC > TT tandem mutations at −8_9 bp were present in 17 tumors (8.9%). Additional mutations included two C > T transitions at −12 bp, and two C > A transversions at −9 bp. The C > T mutation at −12 bp in two BCCs co-occurred with −8C > T and −8/−9CC > TT mutations, respectively, and the C > T mutation at −13 bp in one BCC co-occurred with −8/−9 CC > TT (Table 1**;** Supplementary Table 1).

### Association of mutations with patient and tumor characteristics

Overall, 28 (14.7%) tumors carried alterations at all four loci; 37 (19.4%) tumors carried alterations at three loci (*PTCH1* and *TERT* promoter along with alteration at either *TP53* or *DPH3* promoter); 32 tumors (16.8%) had alterations in 2 genes (*PTCH1* gene and *TP53* or *TERT* promoter or *DPH3* promoter); 14 (7.3%) tumors had only *PTCH1* mutations, while 42 (22.0%) tumors that were wild type for *PTCH1* and carried any of the other alterations **(**Fig. 1**)**. *PTCH1* mutations tend to co-occur with: *TP53* mutations (OR 7.69; 95% CI 3.38–17.46; *P* < 0.0001), *TERT* promoter mutations (OR 3.84; 95% CI 2.08–7.07; *P* < 0.0001) and *DPH3* promoter alterations (OR 5.09; 95% CI 2.56–10.12; *P* < 0.0001) (Fig. 1). No mutation was detected in any of the surrounding normal skin tissue corresponding to a tumor from the same patient.

**Figure 2:**
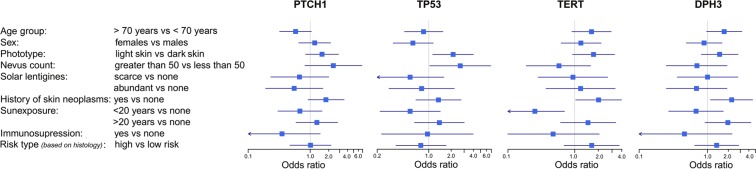
Association between mutations and tumor/patient characteristics: Forest plot were plotted to depict OR and 95%CI for the associations analyzed through univariate logistic regression.

The presence of *PTCH1* mutations tended to associate with decreased patient age at diagnosis (OR 0.58, 95%CI 0.32–1.04, P 0.07); *TP53* mutations associated statistically significantly with light skin color (OR 2.13, 95%CI 1.13–4.00; P 0.02) and>50 nevus count (OR 2.66, 95%CI 1.03–6.87, P 0.04). Non-coding mutations in *TERT* promoter (OR 2.02, 95%CI 1.03–3.97, P 0.04) and *DPH3* promoter mutations (OR 2.25, 95%CI 1.10–4.57, P 0.03) were associated with history cutaneous neoplasms (Fig. 2**;** Supplementary Table 5**)**. In the multivariate analysis, the association between *TP53* mutations and fair skin (OR 5.31, 95%CI 2.19–12.88; P 0002); *DPH3* promoter mutations and history of cutaneous neoplasm (OR 2.47, 95%CI 1.06–5.72; P 0.04) remained statistically significant. In a separate multivariate analysis the simultaneous presence of mutations at all four loci in BCC tumors associated with light skin color (OR 4.84, 95%CI 1.09–21.45; P 0.04) and history of cutaneous neoplasms (OR 8.85, 95%CI 1.51–51.80; P 0.02; data not shown).

### *TERT* expression

**Figure 3:**
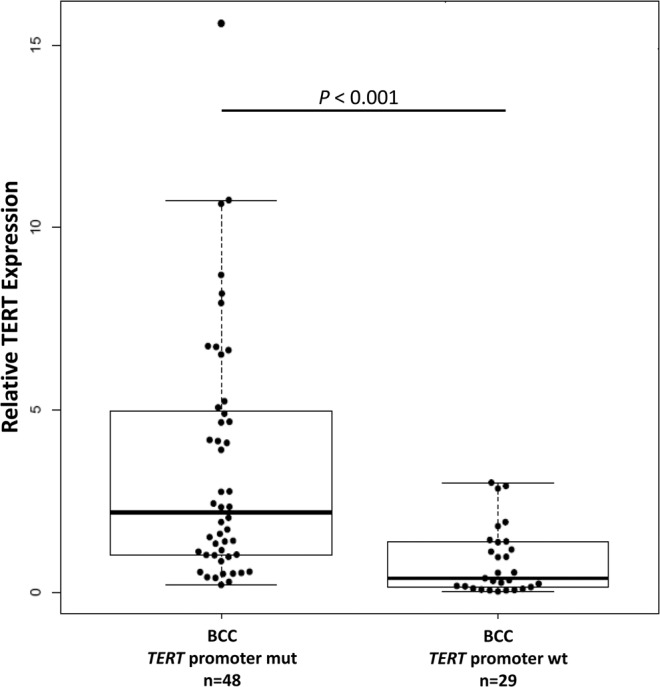
Relative *TERT* expression in BCC tumors based on the promoter mutational status: Experiments were carried out in triplicates and box plots represent mean ± standard error of means; *P*-value was determined by *t*-test.

RNA was available from 77 BCC tumors and corresponding tumor-surrounding skin. Of 77 tumors, 48 carried *TERT* promoter mutations, 34 with −146C > T, 8 with −124C > T, 6 with −138_139CC > TT and 29 were without mutations. Data analysis showed statistically significantly higher mRNA levels in BCC tumors with *TERT* promoter mutations than in tumors without mutations (*P* < 0.001, *t*-test; Fig. 3). Further stratification showed that tumors with *TERT* promoter mutations and complete methylation (n = 23) had statistically significantly (*P* = 0.003) higher *TERT* expression than the tumors with complete methylation and without *TERT* promoter mutations (n = 14) (Supplementary Fig. 3). Similarly, the tumors with partial methylation and *TERT* promoter mutations (n = 25) had statistically significantly (*P* = 0.004) higher *TERT* expression than corresponding tumors with partial methylation and without the *TERT* promoter mutations (n = 2). The difference in *TERT* expression in tumors based on only methylation status was not statistically significant (*P* = 0.82).

### Telomere length

Results from the measurement of relative telomere length were available for 174 BCCs and 104 tumor-surrounding skin tissues. Of 174 BCCs, 100 were with and 74 were without *TERT* promoter mutations. Relative telomere length ranged between 0.19 and 3.62 with median values of 1.02 in tumor-surrounding skin, 0.81 in tumors without *TERT* promoter mutations and 0.72 in tumors with the promoter mutations. Tumors had shorter telomeres than the surrounding corresponding skin tissues. Telomere with *TERT* promoter mutations had shorter telomeres than the tumors without those mutations; however, the difference was not statistically significant (Supplementary Fig. 4).

## Discussion

In this study, other than confirming the high frequency of *PTCH1* and *TP53* mutations in BCC tumors, we showed recurrent noncoding mutations within the *TERT* and *DPH3* promoters. *TERT* promoter mutations were the most frequent alterations followed by *PTCH1* mutations; the simultaneous occurrence of mutations in the four investigated loci was more frequent than per chance. Tumors with *TERT* promoter mutations had higher transcription levels of telomerase reverse transcriptase subunit than the lesions without those alterations. We observed no statistically significantly increase in *TERT* transcription levels in tumors due to an increased methylation within the THOR.

Inactivation of *PTCH1* through mutations confers Sonic Hedgehog independent growth, genomic instability and tumor development potential. The full-length human *PCTH1* consisting of 1447 amino-acids and 23 exons is predicted to contain 12 transmembrane segments, two extracellular domains and two intracellular domains^32,33^. The prevalent mutations in *PTCH1* block protein maturation and consequent abolition of its function but the degree of impairments is dependent on the mutation-type^34,35^. In this study, we observed inactivating alterations in *PTCH1* in 58.6% of tumors. In accordance with a central role of UV radiation in the pathogenesis of BCC, the majority of point mutations were represented by C > T and CC > TT transitions as also reported in previous studies^9,36,37^. The observed frequency of mutations and loss of heterozygosity at the *PTCH1* locus in this study was similar to earlier studies^38^. However, the mutation frequency was lower than that reported in an exome sequencing based genomic analysis^8^.

Approximately 31% of tumors carried mutations in the *TP53* gene and similar to *PTCH1*, a majority of those alterations reflected UV etiology. Most of the base changes in *TP53* were predominantly protein altering missense mutations and to lesser extent nonsense alterations. The mutations mainly affected the DNA binding domain with typical hotspot mutations as reported earlier^8^. The selection for missense mutations within the DNA binding domain of the gene is driven through dominant negative effect where mutant forms hinder functioning of the protein from intact alleles^16^. The observed frequency of the mutations in our study was closer to that reported in non-aggressive than in aggressive BCC tumors^38,39^. However, in an exome sequencing study on a large series of BCC tumors, the frequency of *TP53* mutations was about 61%^9^. The frequencies of mutations detected in BCC tumors at known hotspots like codons 177, 196, 245, 248, 258 and 278 in *TP53* were in conformity with earlier reports^18,19^. Impaired p53 in mice has been shown to result in lack of tanning response and addiction to sunlight; in our data, we observed a strong association between *TP53* mutations and fair skin^13,40^.

*TERT* promoter mutations were detected in 59.2% of tumors and in accordance with earlier reports it was the most frequently altered locus in BCC^23–25^. Of the two main *TERT* promoter mutations, −124 C > T and −146 C > T, the former, with the exception of skin neoplasms, is overwhelmingly predominant in most cancers^22^. However, in melanoma and keratinocyte cancers, the −146 C > T is the most frequent *TERT* promoter mutation^22^. Skin cancers are also characterized by the presence of CC > TT tandem mutations at −124/−125 and −138/−139 bp positions that lead to the similar CCGGAA consensus binding site for ETS transcription factors^41^. The −138/−139 CC > TT tandem mutation in melanoma was shown to be associated with the worst melanoma-specific patient survival^42^. The altered site due to the −146 C > T mutation specifically involves non-canonical NF-kB signaling with cooperative binding between p52/RelB and ETS1 rather than general binding of the multimeric GABPA/GABPB1 complex as reported in gliobastoma, liver and bladder cancer cell lines^29,43–45^. In accordance with previous studies, we detected increased transcription of the reverse transcriptase subunit in BCC tumors with than without the *TERT* promoter mutations^46,47^.

We also investigated the THOR region where methylation of CpG sites is generally associated with increased *TERT* expression^30,48^. However, our data show that increased *TERT* expression in BCC tumors was primarily attributed to the promoter mutations similar to adult gliomas^49^. Telomeres in tumors with the *TERT* promoter mutations are usually shorter than in tumors without those mutations as observed in melanoma and gliomas^47,50^. Though, we did observe that telomere were shorter in BCC tumors with the promoter mutations than in tumors without those mutations, the difference was not statistically significant. The decreased telomere length in tumors with somatic *TERT* promoter mutations signifies delayed effect in stabilization of telomeres until after generation of genomic instability^22,51^.

Noncoding mutations within the *DPH3* promoter were detected in 38.2% of the lesions. The effect of the mutations, located within the proximity to an ETS binding motif, has been rather ambiguous^31^. The presence of mutations exclusively at dipyrimidinic sites coupled with typical CC > TT tandem alterations in the *DPH3* promoter again signified the role UV radiation through sun-exposure. In the absence of a definite functionality, the *DPH3* promoter mutations being mere passenger events cannot be ruled out^52^. However, we did observe that the *DPH3* mutations associated with an increased risk of cutaneous neoplasms. *DPH3* encodes a short peptide involved in electron transfer during the synthesis of eukaryotic diphthamide and forms a complex with Kti13, which functions in both tRNA and translational elongation factor 2 (EF2) modifications^53,54^. Overexpression of *DPH3* has been shown to promote migratory ability of murine melanoma and downregulation of its expression was shown to inhibit cellular invasion and metastasis *in vivo*^55,56^.

In conclusion, we described noncoding mutations within the *TERT* and *DPH3* promoter at high frequency in BCC tumors in addition to frequent alterations in *PTCH1* and *TP53* genes that impair protein functions. Although, the mutations in *PTCH1*, *TP53*, and *TERT* promoter have been reported earlier, this is the first communication to report alterations in those loci and the *DPH3* promoter simultaneously in BCC tumors and to describe methylation levels within the THOR and transcription of the telomerase reverse transcriptase subunit^23–25^. Interestingly, the alterations in *PTCH1*, *TP53* and *DPH3* promoter occurred more frequently in tumors with *TERT* promoter mutations. The simultaneous presence of the mutations in four loci associated with fair skin and history of cutaneous neoplasms. It is likely that the increased cellular proliferation following the activation of Hedgehog signaling or elimination of checkpoints due to p53 loss would require telomere buttressing due to increased cellular proliferation, which is probably attained through telomerase rejuvenation via *TERT* promoter mutations. The *TERT* promoter mutations impart a distinctive tumor environment characterized by epithelial-to-mesenchymal transition (EMT) gene expression signature and MAPK signaling^57^. Although, BCC tumors rarely metastasize; a small but significant proportion of tumors progress to an invasive disease with metastasis resulting in patient mortality. Inoperable patients with metastatic disease are treated with vismodegib and sonidegib that impede Hedgehog pathway through inhibition of SMO^58^. Patients with basal cell nevus syndrome respond to the targeted treatment with a low rate of acquired resistance^12^. It is likely the *TP53* and *TERT* promoter mutations could impact the treatment outcome.

## Material and Methods

### Patients and tissues

Fresh-frozen BCC tumor tissues from 191 patients were included in this study. Of those patients 191 patients, corresponding normal appearing skin from 115 patients was available and included in the study **(Supplementary information;** Supplementary Table 1**)**. Seventy-one (37.2%) nodular, 42 (22.0%) superficial, and 4 (2.1%) adenoid tumors were grouped as low risk. Twenty-four (12.6%) infiltrative, 7 (3.7%) were morphoeic, 6 (3.1%) micronodular, and one metatypical type were categorized as high risk. For 36 (18.9%) tumors histological data were not available. BCC lesions were retrieved from the Biobank of the Instituto Valenciano de Oncología in Valencia, Spain and collected at the Department of Dermatology of the University of L’Aquila, Italy. The study was approved by the Instituto Valenciano de Oncología Ethics Committee and all methods were performed in accordance with the relevant guidelines and regulations. A written informed consent was signed by all study participants.

### Mutational Analysis

DNA and total RNA were extracted from fresh-frozen tissues using the QIAGEN AllPrep DNA/RNA/miRNA Universal Kit (QIAGEN, Hilden, Germany) according to the manufacturer’s instruction.

Mutations at different loci were screened using PCR and Sanger sequencing (**Supplementary information;** Supplementary Table 6). For *PTCH1* a set of 21 pairs of primers flanking from exon 3 to exon 24 of the *PTCH1* gene was used and *TP53* was sequenced for mutations in exons 5, 6, 7, 8 and 9. For *TP53* and *PTCH1* each PCR was carried out in 10 µl volume containing 10 ng DNA, 0.11 mM dNTP and 0.15 µM of each primer and, 5-µl HotStar Taq Plus Master mix kit (Qiagen). These regions presented a high frequency of mutations as reported in COSMIC database for the transcript ENST00000269305 that encodes the longest isoform. For *TERT* promoter, PCR was carried out in a 10-µl volume containing 10 ng DNA, 50 mM KCl, 0.11 mM dNTP, 2 mM MgCl_2_ and 0.11 mM of each primer and Taq polymerase (GENAXXON biosciences GmbH). PCR for *DPH3* was carried out in a 10-µl volume containing 10 ng DNA, 25 mM MgCl_2_, 0.11 mM dNTP, 5% DMSO and 0.11 mM of each primer and Taq polymerase (GENAXXON biosciences GmbH). Denaturation temperatures for PCR were set at 95 °C for 45 sec, annealing temperature were adjusted according to primer sequences, and polymerization at 72 °C for 30 sec each for 35 cycles. Amplified products were purified with ExoSAP (Illustra ExoProStar, GE Healthcare Life Sciences) and were subjected to 35 cycles of sequencing reaction with a di-deoxy terminator kit (BigDye Terminator v3.1 Cycle Sequencing Kit, life technologies, Thermo Fisher Scientific Inc.) and analyzed in a capillary sequencer (AbiPrism 3130xl Genetic Analyzer).

Sequencing data were analyzed using Geneious Pro 5.6.5 software and sequences from the NCBI gene database were used as references, *PTCH1* (chr9: 98,205,262-98,279,339 hg 19 coordinates), *TP53* (chr17: 7,565,097-7,590,856 hg19 coordinates), *TERT* promoter (chr5: 1,295,071-1,295,521, hg 19 coordinates), and *DPH3* promoter (chr3: 16,306,256-16,306,755, hg19 coordinates) (Supplementary Fig. 5). The point mutations in the *PTCH1* and *TP53* genes were annotated by using web-based tool Mutalyzer and variant effector predictor (VEP) algorithm from Ensembl database^59^. The sequence topology for the *PTCH1* gene was generated with PROTTER^60^. Mutations with intron/exon boundaries were analyzed for the effect on splicing using maximum entropy model^61^. For splice site analysis on the 5′ end, three nucleotides from exon and 6 nucleotides from the following intron were included in the model; for 3′ splice site, 20 nucleotides from the intron and three nucleotides from the preceding exon were included in the model.

### Multiplex ligation-dependent probe amplification (MLPA)

MLPA method with specific probes was used (SALSA MLPA P067-B2 *PTCH1* probemix, MRC-Holland, Amsterdam, The Netherlands) to detect deletions/duplications in the *PTCH1* gene. The results were cross-validated by Sanger sequencing and concordant results were confirmed by both methods **(**Supplementary Fig. 5**)**.

### Measurement of TERT promoter methylation

A 213 bp genomic region within the *TERT* promoter, from −560 to −774 (chr5:1,295,665-1,295,878; hg19) from ATG start site was screened for CpG sites in-silico using MethPrimer^62^. The selected region included 14 CpG sites and primers were designed to amplify both methylated and unmethylated sequences. PCR product was either sequenced directly or after cloning into T-overhang vector. The sequence data files were further analyzed for CpG methylation status using a web-based bisulfite sequencing analysis tool called QUMA (QUantification tool for *Methylation* Analysis) under default settings^63^.

### Measurement of TERT mRNA expression by quantitative real-time PCR (qRT-PCR)

For measurement of gene expression, each reverse transcription reaction was performed using approximately 1.0 µg RNA and random hexamer primers using a cDNA synthesis kit (Thermo Scientific, Waltham, USA). The real-time PCR was carried out in triplicates on a 384-well layout using primers specific for *TERT* (Supplementary Table 6) and primers for the *GUSB* gene (Qiagen). Difference in gene expression levels were calculated following the ΔΔC_T_ method; GUSB expression was used as an internal reference (ΔC_T_) and difference in expression levels was calculated between BCCs and matched tumor-surrounding skin (ΔΔC_T_) followed by performing a log_2_ transformation.

### Measurement of telomere length

Relative telomere length in tumor DNA was measured using the monochrome multiplex PCR assay as described previously including minor modifications^64–66^. The standard curve was used to quantify the telomere (T) and albumin genes (S) based on the respective average Ct values obtained in triplicate. The relative telomere length was expressed as the ratio between T/S. Inter-assay and intra-assay variation were determined by duplicating the reference DNA for all the dilutions in all the assays performed.

### Statistics

The associations between mutations in *PTCH1*, *TP53*, *TERT* promoter, *DPH3* promoter and patient age at diagnosis, sex, phototype, nevus count, solar lentigos, history of cutaneous neoplasms, exposure to sun, immunosuppressive treatment and histology were determined by χ^2^-tests and size of the effect determined by odds ratio (OR) and 95% confidence intervals (CIs) in logistic regression model. Multivariate logistic regression was also carried and included statistically significant variables from univariate analysis. Box plots were drawn to for *TERT* expression and telomere length in tumors with and without *TERT* promoter mutations and differences analyzed by two-tailed t-test. Similar box-plots were also drawn after stratification of tumors based on THOR methylation status.

## Supplementary information

Supplementary information.

Supplementary Dataset 1.
