# Supplementary material for: Coding and noncoding somatic mutations in candidate genes in basal cell carcinoma
Source: Sci Rep. 2020 May 14;10:8005. doi: 10.1038/s41598-020-65057-2 (PMC7224188; doi:10.1038/s41598-020-65057-2)
Supplement: Supplementary file 1 — Supplementary information. [file 41598_2020_65057_MOESM1_ESM.pdf]

Supplementary Information

Coding and noncoding somatic mutations in candidate genes in basal cell carcinoma

Maria Giovanna Maturo[1], Sivaramakrishna Rachakonda[2,3], Barbara Heidenreich[3], Cristina Pellegrini[1], Nalini Srinivas[2], Celia Requena[4], Carlos Serra-Guillen[4], Beatriz Llombart[4], Onofre Sanmartin[4], Carlos Guillen[4], Lucia Di Nardo[5], Ketty Peris[5], Maria Concetta Fargnoli[1], Eduardo Nagore[4], Rajiv Kumar[2,3]

[1]Department of Dermatology, Department of Biotechnology and Applied Clinical Sciences, University of L'Aquila, L'Aquila, Italy

[2]Division of Functional Genome Analysis, German Cancer Research Center, Heidelberg, Germany

[3]Division of Molecular Genetic epidemiology, German Cancer Research Center, Heidelberg, Germany

[4]Department of Dermatology, Instituto Valenciano de Oncología, València, Spain

[5]Institute of Dermatology, Catholic University of Sacred Heart, Fondazione Policlinico Universitario A. Gemelli, IRCCS, Rome, Italy

Running title: Mutations in BCC

Key words: Basal cell carcinoma, *TERT* promoter, *DPH3*, PTCH1, mutations

*Correspondence to:

Rajiv Kumar

Division of Functional Genomic Analysis

German Cancer Research Center

Heidelberg, Germany

Email: r.kumar@dkfz.de

Methods

Patients and tissues

Demographic and clinical characteristics of patients including age, sex, skin type, sun exposure, nevus count, solar lentigines, other skin cancers, immunosuppression, anatomical site and histological subtype of tumors were registered through a standardized questionnaire, skin examination and medical records **(Supplementary Table 1)**. During surgical excision of the lesions, a 4 mm intra-tumoral punch biopsy specimen was obtained, snap-frozen in liquid nitrogen and kept at -80°C until nucleic acid extraction. The remaining tissue was formalin-fixed and paraffin-embedded (FFPE) for conventional histopathology.

Mutational Analysis

Sequencing data were analyzed using Geneious Pro 5.6.5 software and sequences from the NCBI gene database were used as references, *PTCH1* (chr9: 98,205,262-98,279,339 hg 19 coordinates), *TP53* (chr17: 7,565,097-7,590,856 hg19 coordinates), *TERT* promoter (chr5: 1,295,071-1,295,521, hg 19 coordinates), and *DPH3* promoter (chr3: 16,306,256-16,306,755, hg19 coordinates) (**Supplementary Figure 5**). The point mutations in the *PTCH1* and *TP53* genes were annotated by using web-based tool Mutalyzer and variant effector predictor (VEP) algorithm from Ensembl database[1]. The sequence topology for the *PTCH1* gene was generated with PROTTER[2]. Mutations with intron/exon boundaries were analyzed for the effect on splicing using maximum entropy model[3]. For splice site analysis on the 5' end, three nucleotides from exon and 6 nucleotides from the following intron were included in the model; for 3' splice site, 20 nucleotides from the intron and three nucleotides from the preceding exon were included in the model.

1 Wildeman, M., van Ophuizen, E., den Dunnen, J. T. & Taschner, P. E. Improving sequence variant descriptions in mutation databases and literature using the Mutalyzer sequence variation nomenclature checker. *Human mutation* **29**, 6-13, doi:10.1002/humu.20654 (2008).
2 Omasits, U., Ahrens, C. H., Muller, S. & Wollscheid, B. Protter: interactive protein feature visualization and integration with experimental proteomic data. *Bioinformatics (Oxford, England)* **30**, 884-886, doi:10.1093/bioinformatics/btt607 (2014).
3 Yeo, G. & Burge, C. B. Maximum entropy modeling of short sequence motifs with applications to RNA splicing signals. *Journal of computational biology : a journal of computational molecular cell biology* **11**, 377-394, doi:10.1089/1066527041410418 (2004).

Legends to Supplementary Figures

Supplementary Figure 1. Schematic representation of the *PTCH1* gene using PROTTER; all mutations identified in the BCC tumors are highlighted manually.

Supplementary Figure 2. Methylation pattern at 14 CpG sites at the *TERT* promoter (chr5:1,295,665-1,295,878; hg19 coordinates) determined by bisulfite modification, cloning, sequencing and analysis using QUMA. The upper panel represents methylation levels in 10 tumors without the lower panel represents methylation levels in 10 tumors with the *TERT* promoter mutations. Each circle represents a CpG site with corresponding genomic location at the top. Black colored region of the circles denotes methylated (percent) and the light striped region denotes unmethylated (percent). The statistical difference in methylation patterns is shown underneath each CpG site.

Supplementary Figure 3. Relative *TERT* gene expressions in BCCs with and without *TERT* promoter mutations based on *TERT* methylation status: Experiments were carried out in triplicates and box plots represent mean ± standard error of means; *P*-value was determined by *t*-test.

Supplementary Figure 4. Telomere lengths in BCC tumors and surrounding skin: Relative telomere length in surrounding skin, in BCC without and with *TERT* promoter mutations. Experiments were carried out in triplicates and box plot represent mean ± S.E. *P*-value was determined by *t*-test.

Supplementary Figure 5. Representative sequencing electropherographs: Frequent mutations detected in BCC tumors in **A)** *PTCH1,* **B)** *TP53,* **C)** *TERT* promoter and **D)** *DPH3* promoter are depicted. In the top panel with representative *PTCH1* sequences, heterozygous mutations are shown. In the bottom panel with representative *PTCH1* sequences, homozygous deletions are shown in two tumors, which also showed loss of heterozygosity in MLPA.

Supplementary Figure 1

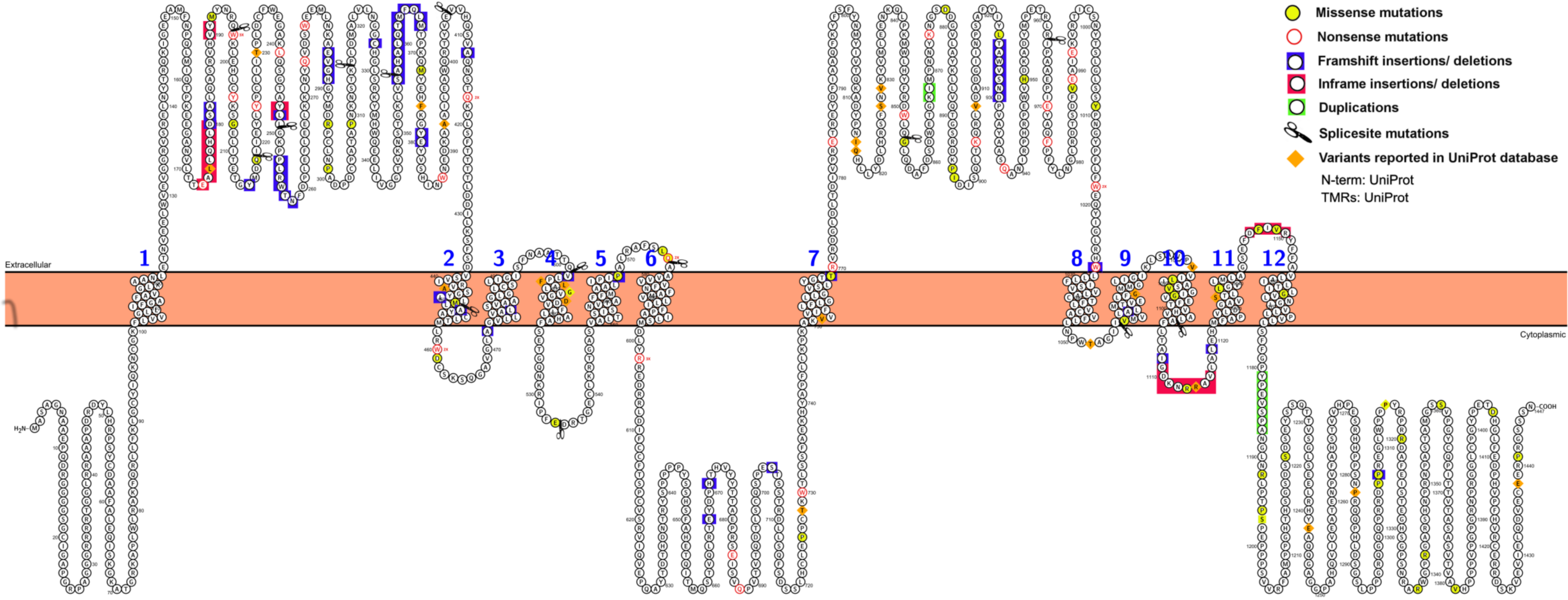

Supplementary Figure 2

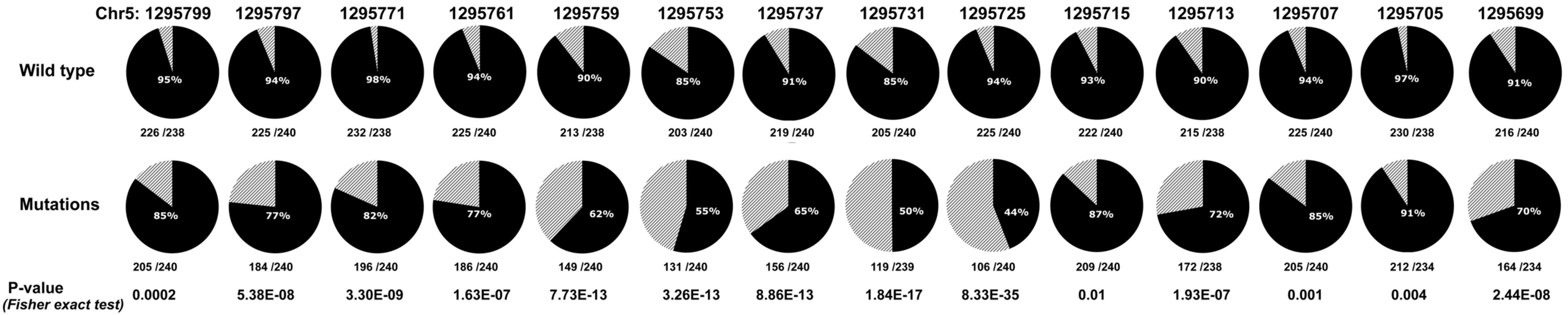

Supplementary Figure 3

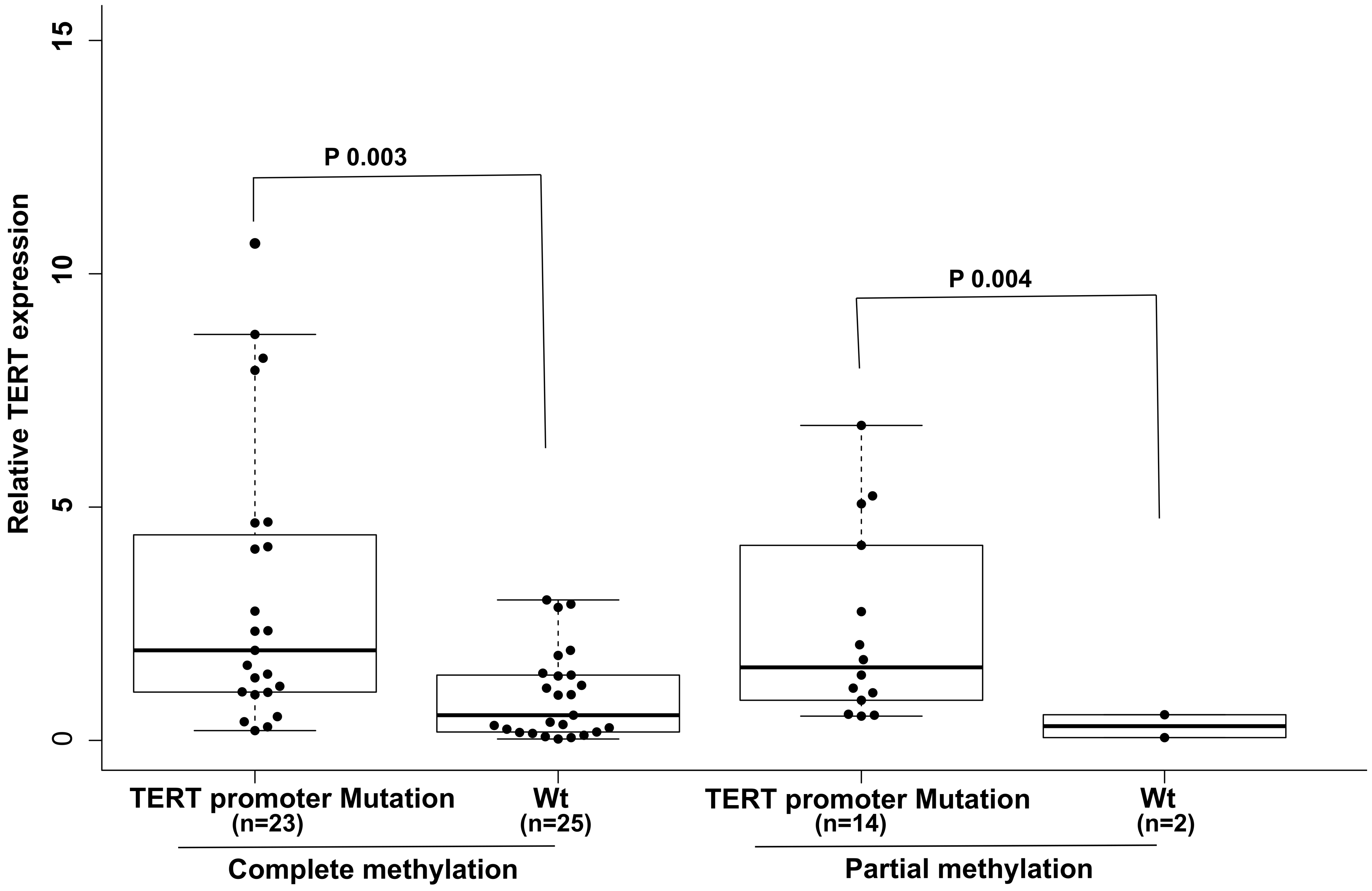

Supplementary Figure 4

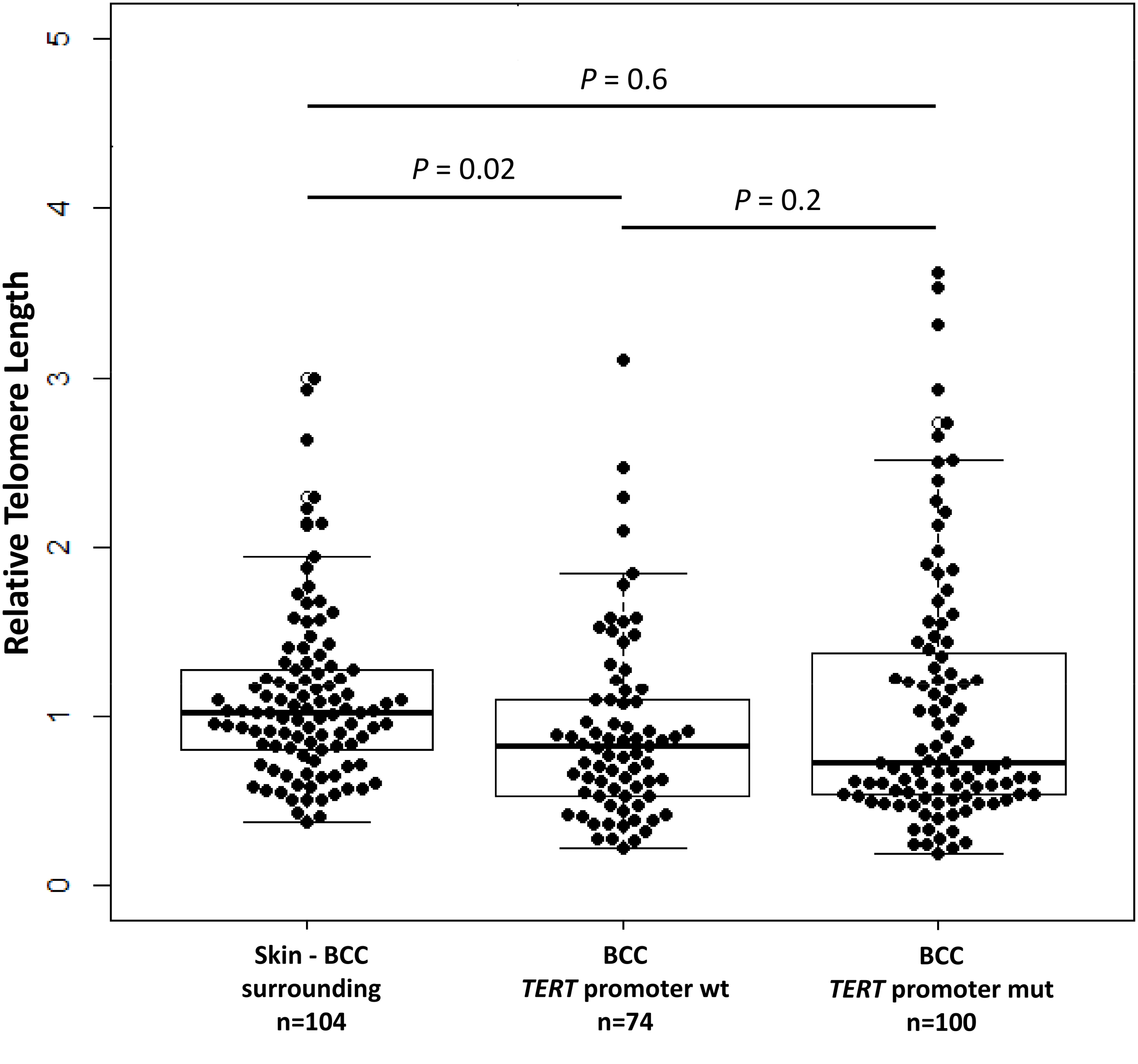

Supplementary Figure 5

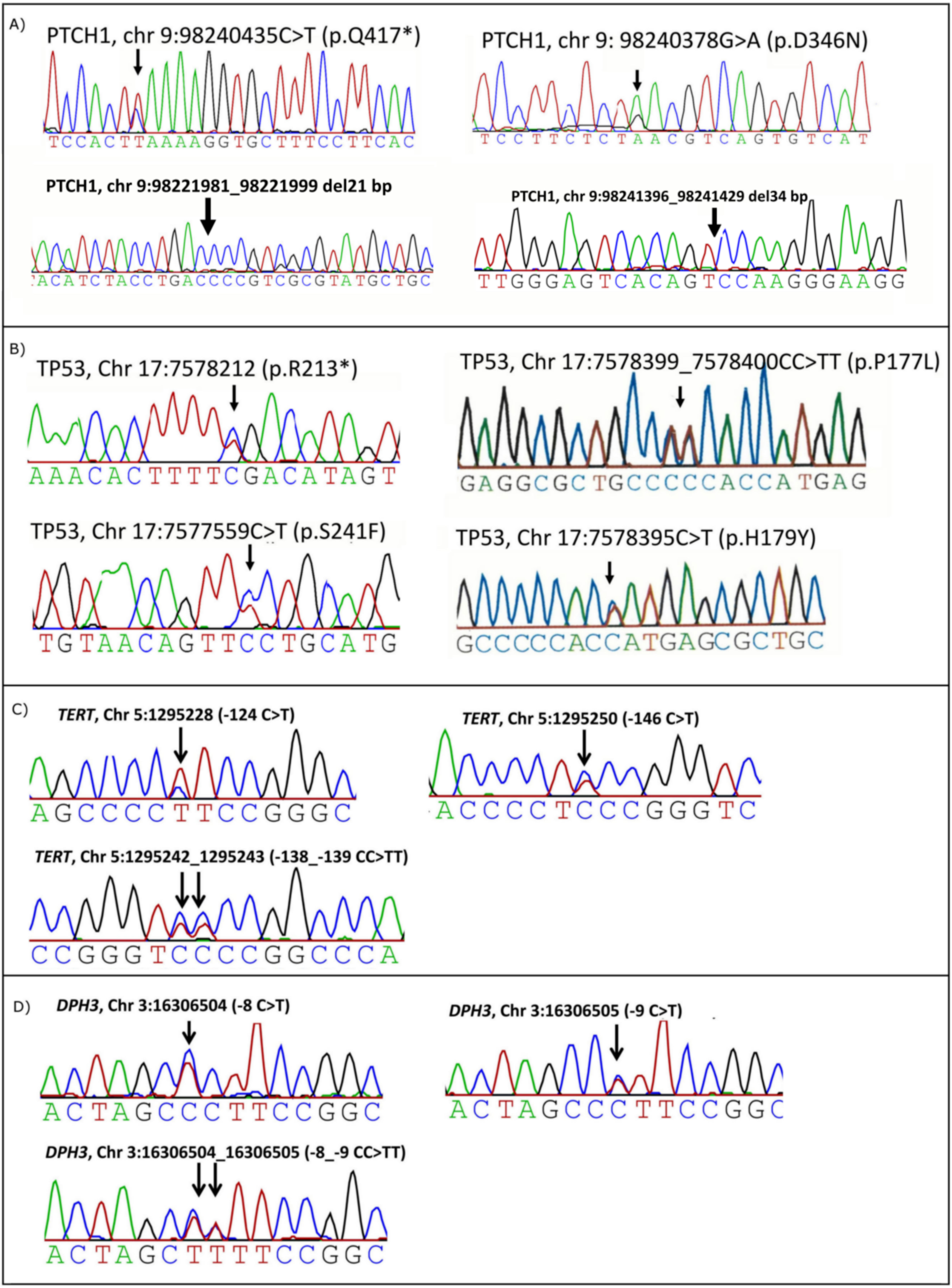

**Supplementary Table 2: PTCH1 mutations in BCC tumors**						
**Tumor-ID**	**Genomic position (NC_000009.11)**	**Exon /Intron**	**CDS mutation (NM_000264.3)**	**Amino acid mutation (CCDS6714)**	**COSMIC database**	**MLPA**
1	g.98240435	Exon 9	c.1249C>T	p.Q417*	p.Q417H	LOH
2	g.98215760	Exon 20	c.3449C>T	p.G1149G		LOH
4	g.98238318_98238319	Exon 12	c.1725_1726CC>TT	p.L575L; Q576*		LOH
6	g.98240469	Intron 8	c.1216-1G>A		c.1216-1G>A	
8	g.98240337	Exon 9	c.1347G>A	p.M449I	p.M449I	LOH
	g.98244231_98244239	Exon 5	c.738_746del9	p.Y247_L249del		
10	g.98209217	Exon 23	c.4321C>T	p.P1441S	p.P1441S	LOH
	g.98229650_98229651	Exon 15	c.2307_2308CC>TT	p.T769T; R770*	c.2307_2308CC>TT	
11	g.98215760	Exon 20	c.3449C>T	p.G1149G		
13			.			LOH
15	g.98232213_98238316	Exon 12/ Intron 12	c.1728G>A;c.1728-1G>A	p.Q576Q		LOH
18	g.98209514	Exon 23	c.4024C>G	p.R1342G		
	g.98239951_98239952	Exon 10	c.1380_1381GG>AA	p.W460*; D461N		
	g.98215854	Exon 20	c.3355delC	p.L1119fs*20		
21	g.98239140	Intron 10	c.1504-1T>C			
22	g.98239140	Intron 10	c.1504-1T>C			
23	g.98212185	Exon 21	c.3487G>A	p.G1163S	p.G1163S	
24	g.98231093_98231094	Exon 14	c.2189_2190GG>AA	p.W760*		
26	g.98220292	Intron 18	c.3168+3C>G			
27	g.98240435	Exon 9	c.1249C>T	p.Q417*	p.Q417H	
30	g.98220409	Exon 18	c.3054G>A	p.W1018*	p.W1018*	
31	g.98229405_98229406	Exon 15	c.2552_2553GG>AA	p.W851*	p.W851*	
32	g.98244322_98244416	Exon 4/ Intron 4	c.654G>A;c.654-1G>A	p.Q218Q	p.Q218Q	LOH
33	g.98239918	Exon 10	c.1414delGinsTT	p.A472fs*25		LOH
35	g.98242735	Exon 6	c.882C>T	p.R294R		

42	g.98239896	Exon 10	c.1436delT	p.L479fs*12		LOH
43	g.98242671	Intron 6	c.945+1C>T			LOH
44	g.98244305	Exon 5	c.672C>A	p.Y224*	p.Y224fs*4	LOH
45	g.98224280	Exon 15	c.2561G>A	p.G854R	p.G854R	
	g.98231282	Exon 14	c.2001delGTACinsTTA	p.E667fs*2		
46	g.98209458	Exon 23	c.4080C>T	p.S1360S	p.S1360S	
	g.98242670	Intron 6	c.945+2T>A			
48	g.98241378	Exon 8	c.1119C>T	p.Y372Y		
49	g.98218607	Exon 19	c.3257T>C	p.L1086P		LOH
51	g.98211581	Exon 22	c.3574C>T	p.R1192C		
53	g.98218602_98218627	Exon 19	c.3237_3262dup21	p.V1081_I1087dup		
54			.			LOH
61			.			LOH
62	g.98209397	Exon 23	c.4141G>A	p.V1381M	p.V1381M	
63	g.98231172_98231173	Exon 14	c.2110_2111delAG	p.S704fs*33		LOH
64	g.98231221	Exon 14	c.2062C>T	p.Q688*	p.Q688*	LOH
65	g.98221921	Exon 17	c.2848C>T	p.H950Y		Focal deletion (exons_4-5-6-7-8)
66	g.98238318_98238319	Exon 12	c.1725_1726CC>GT	p.L575L; Q576*		LOH
	g.98229644_98229652	Exon 15	c.2306_2314insG	p.T769fs		
67	g.98232138	Exon 13	c.1804C>T	p.R602*	p.R602*	
69	g.98244479	Exon 4	c.591G>A	p.W197*	p.W197*	
	g.98240474	Intron 8	c.1216-6C>A		c.1216-6C>A	
	g.98224136	Intron 16	c.2703+2T>G			
	g.98209616_98209617	Exon 23	c.3921_3922insC	p.P1307fs*17	c.3921_3922insC	
70	g.98224152	Exon 16	c.2689A>G	p.I897V		
71	g.98242671	Intron 6	c.945+1C>T			LOH
72	g.98220293	Intron 18	c.3168+2T>A			

73	g.98218682	Exon 19	c.3182delCGCinsTG	p.A1061Vfs*1		
75	g.98247980_98247982	Exon 3	c.569_571delTAT	p.V190_Y191del		
78	g.98209217	Exon 23	c.4321C>T	p.P1441S	p.P1441S	
80	g.98224237_98224242	Exon 16	c.2599_2604insAAATC	p.K2899_I2900dup		
81	g.98239117	Exon 11	c.1526G>A	p.G509D	p.G509D	
84	g.98220410	Exon 18	c.3053G>A	p.W1018*	p.W1018*	LOH
85	g.98244451	Exon 4	c.619G>A	p.G207R		LOH
86	g.98242783	Exon 6	c.834G>A	p.W278*	p.W278*	
90	g.98242752_98242762	Exon 6	c.855_865del 11	p.E286_H289delfs*30		
91	g.98241336	Exon 8	c.1161G>A	p.W387*	p.W387*	Focal deletion (exons_18-19)
92	g.98248006_98248012	Exon 3	c.539_545delACTCGGC	p.D180_A182delfs*37		
	g.98221878	Intron 17	c.2887+4del AGTA			
93	g.98239952	Exon 10	c.1380G>A	p.W460*		
	g.98220436	Exon 18	c.3027C>T	p.Y1009Y	p.Y1009Y	
94	g.98209325	Exon 23	c.4213G>A	p.D1405N		
	g.98218695_98220295	Exon 19 /Intron 19	c.3168G>A;c.3168-1G>A	p.V1057M		
95	g.98218587	Exon 19	c.3277G>A	p.G1093R		LOH
98	g.98238341	Exon 12	c.1703C>T	p.P568L	p.P568L	LOH
	g.98240344	Exon 9	c.1340delA	p.L447Tfs*9		
99	g.98221981_98221999	Exon 17	c.2770_2788del21	p.T924Cfs*32		
100	g.98240378	Exon 9	c.1306C>T	p.D436N		
101	g.98211568	Exon 22	c.3587C>T	p.P1196L		
101	g.98220541_98220542	Exon 18	c.2921_2922insAGTT	p.F974*	p.F974C	
102	g.98211564	Exon 22	c.3591C>T	p.S1197S	p.S1197S	Focal deletion (From exon_7 to exon_25)
	g.98224281	Intron 15	c.2561-1G>A		c.2561-1G>A	
104	g.98224221	Exon 16	c.2620A>T	p.K874*		

105	g.98221957	Exon 17	c.2812C>T	p.Q938*	p.Q938*	
106	g.98220382_98220383	Exon 18	c.3080_3081CC>TT	p.W1027*	p.W1027*	LOH
107	g.98231110	Exon 14	c.2173C>T	p.P725S	p.P725S	
	g.98238441_98239041	Exon 11/ Intron 11	c.1602C>T;c.1602-1C>T	p.E534N		
	g.98215886_98215887	Exon 20	c.3322_3323insA	p.I1108Nfs*37		
109	g.98241361	Exon 8	c.1136delTCGT	p.Y379_E380delfs*52		
110	g.98240335	Intron 9	c.1347+2C>T			LOH
111	g.98244480	Exon 4	c.590G>A	p.W197*	p.W197*	LOH
113	g.98211493	Exon 22	c.3662C>T	p.S1221F	p.S1221F	LOH
114	g.98241429_98242251	Intron 7	c.1067_1068insA			
115	g.98242721	Exon 6	c.896C>T	p.P299L		LOH
	g.98222001	Exon 17	c.2768T>C	p.L923P	p.L923P	
	g.98209593	Exon 23	c.3945C>T	p.P1315P		
119	g.98232138	Exon 13	c.1804C>T	p.R602*	p.R602*	
124	g.98232138	Exon 13	c.1804C>T	p.R602*	p.R602*	
125	g.98244236_98244237	Exon 5	c.740_741insC	p.L248Pfs*4		
127			.			LOH
128	g.98242797	Exon 6	c.820C>T	p.Q274*		
130	g.98244458	Exon 4	c.612delC	p.Y204*		
131	g.98215760_98215771	Exon 20	c.3438_3449del12	p.D1146_R1150del		
132	g.98244479_98244480	Exon 4	c.590_591GG>AA	p.W197*	p.W197*	
134	g.98224207	Exon 16	c.2634C>T	p.D878D	p.D878D	
	g.98244485	Intron 3	c.585C>T			
138	g.98220434_98220464	Exon 18	c.2999_3029dup30	p.N1000_1009dupfs		LOH
139	g.98220383_98220384	Exon 18	c.3079_3080delTG	p.W1027fs*118		LOH
144	g.98209579	Exon 23	c.3959G>A	p.R1320K	p.R1320K	
	g.98238442	Intron 11	c.1603-1G>A		c.1603-1G>A	
146			.			LOH

147	g.98244423_98244426	Exon 4	c.644_647delACAT	p.Y215fs*5		LOH
148	g.98222059	Exon 17	c.2710A>T	p.K904*	p.K904>IQ	
	g.98209619_98209620	Exon 23	c.3918_3919CC>TT	p.P1306P; P1307S		
150	g.98231233	Exon 14	c.2050G>T	p.E684*	p.E684*	LOH
151	g.98218596	Exon 19	c.3268G>T	p.V1090F		Focal deletion (exons_5-6-7-8)
152			.			LOH
153	g.98224154	Exon 16	c.2687C>T	p.P896L		
	g.98242841_98242860	Exon 6	c.757_776dup19	p.P253_N258fs		
156	g.98247975	Exon 3	c.576G>A	p.M192I		
157	g.98240378	Exon 9	c.1306G>A	p.D436N		LOH
	g.98242672_98242674	Exon 6	c.943_945delAAA	p.K315del		
158	g.98248013_98248033	Exon 6	c.518_538del21	p.E172_D180del		LOH
160	g.98241396_98241429	Exon 8	c.1068_1101del34	p.S356_M367delfs*38		LOH
161	g.98242337	Exon 7	c.981delT	p.C327fs*15		
	g.98239981_98239982	Exon 10	c.1350_1351insG	p.A451Gfs*46		
164	g.98244323	Intron 5	c.655-1G>A		c.655-1G>A	
165	g.98231272	Exon 14	c.2011delC	p.H671Tfs*22		LOH
168			.			LOH
170	g.98220489	Exon 18	c.2974G>T	p.E992*	p.E992*	LOH
171	g.98244255	Exon 5	c.722T>A	p.L241*	p.L241L	
173	g.98220498_98220499	Exon 18	c.2964_2965GG>TT	p.V988V; E989*		
174	g.98242691	Exon 6	c.926C>T	p.P309L	p.P309L	
175	g.98220555	Exon 18	c.2908G>T	p.E970*	p.E970*	LOH
	g.98218532	Intron 19	c.3306+26delTT			
176	g.98240450	Exon 9	c.1234delG	p.A412Hfs*27		
177	g.98209458	Exon 23	c.4080C>T	p.S1360S	p.S1360S	LOH
	g.98215863_98215883	Exon 20	c.3326_3346del21	p.G1109_V1116del		

181	g.98229608	Exon 15	c.2350G>T	p.E784*	p.E784D	
185	g.98209526	Exon 23	c.4012C>T	p.R1338C		LOH
	g.98221881	Exon 17	c.2887+1C>T			
186	g.98242748_98242760	Exon 6	c.857_869del13	p.E286Cfs*34		LOH
187	g.98215768	Exon 20	c.3441C>T	p.F1147F		LOH
	g.98248034	Exon 3	c.517G>T	p.E173*	p.E173V	
188	g.98215871	Exon 20	c.3338G>A	p.R1113H		
	g.98244228	Exon 5	c.746+3A>T			
	g.98238341_98238342	Exon 12	c.1702_1703delCC	p.P568Sfs*58		
189	g.98215805	Exon 16	c.3404A>G	p.L1135P		LOH
190	g.98239128_98239137	Exon 11	c.1506_1515del10	p.V502Vfs*36		

Supplementary Table 3. Splice site mutations in PTCH1 gene					
**5' splice site mutations**					
	CDS location	Sequence	MAX-entropy	Markov Model	PWM
Mutation	c.1728+1G>A	cagATGAGC	MAXENT: 1.42	MM: 2.12	WMM: 2.29
Wild type	c.1728+1G	cagGTGAGC	MAXENT: 9.60	MM: 10.30	WMM: 10.47
Mutation	c.3168+3G>C	attGTCAGT	MAXENT: -0.28	MM: -1.32	WMM: 2.03
Wild type	c.3168+3G	attGTGAGT	MAXENT: 7.35	MM: 5.98	WMM: 5.17
Mutation	c.654+1G>A	cagATATAT	MAXENT: -0.30	MM: -0.94	WMM: -1.51
Wild type	c.654+1G	cagGTATAT	MAXENT: 7.88	MM: 7.24	WMM: 6.68
Mutation	c.945+1G>A	aaaATGAGT	MAXENT: 0.22	MM: -0.40	WMM: -0.02
Wild type	c.945+1G	aaaGTGAGT	MAXENT: 8.40	MM: 7.78	WMM: 8.16
Mutation	c.945+2T>A	aaaGAGAGT	MAXENT: 0.22	MM: -0.40	WMM: -0.02
Wild type	c.945+2T	aaaGTGAGT	MAXENT: 8.40	MM: 7.78	WMM: 8.16
Mutation	c.2703+2T>G	cagGGACTC	MAXENT: -0.61	MM: -1.23	WMM: -2.69
Wild type	c.2703+2T	cagGTACTC	MAXENT: 7.04	MM: 6.41	WMM: 4.96
Mutation	c.3168+2T>A	attGAGAGT	MAXENT: -0.83	MM: -2.20	WMM: -3.01
Wild type	c.3168+2T	attGTGAGT	MAXENT: 7.35	MM: 5.98	WMM: 5.17
Mutation	c.2887+3del	gaaGTAGCG	MAXENT: -3.82	MM: 2.04	WMM: 1.56
Wild type	c.2887+3	gaaGTAAGT	MAXENT: 9.82	MM: 8.09	WMM: 8.70
Mutation	c.3168+1G>A	attATGAGT	MAXENT: -0.83	MM: -2.20	WMM: -3.01
Wild type	c.3168+1G	attGTGAGT	MAXENT: 7.35	MM: 5.98	WMM: 5.17
Mutation	c.1602+1G>A	gagATAATG	MAXENT: 0.55	MM: -1.18	WMM: -1.15
Wild type	c.1602+1G	gagGTAATG	MAXENT: 8.73	MM: 7.00	WMM: 7.03
Mutation	c.1347+2T>C	atgGCAACG	MAXENT: -1.71	MM: -1.64	WMM: -1.86
Wild type	c.1347+2T	atgGTAACG	MAXENT: 6.05	MM: 6.12	WMM: 5.90
Mutation	c.1067+1ins	cagAGTAAG	MAXENT: -12.23	MM: -12.52	WMM: -12.09
Wild type	c.1067+1	cagGTAAGC	MAXENT: 9.88	MM: 11.18	WMM: 11.85
Mutation	c.2887+1C>T	gaaTTAAGT	MAXENT: 1.31	MM: -0.41	WMM: 0.20
Wild type	c.2887+1C	gaaCTAAGT	MAXENT: 1.54	MM: -0.18	WMM: 0.43
Mutation	c.746+3A>T	cctGTTAGT	MAXENT: -5.28	MM: -6.27	WMM: 1.66
Wild type	c.746+3A	cctGTAAGT	MAXENT: 7.52	MM: 6.68	WMM: 6.61

3'splice site mutations					
	CDS location	Sequence	MAX-entropy	Markov Model	PWM
Mutation	c.1216-1G>A	gcattctgttgtgaccacaaGTG	MAXENT: -1.44	MM: -0.36	WMM: -2.01
Wild type	c.1216-1G	gcattctgttgtgaccacagGTG	MAXENT: 7.31	MM: 8.39	WMM: 6.74
Mutation	c.1504-8T>C	atcctctgttttcgctgtagGTT	MAXENT: 10.95	MM: 10.57	WMM: 12.40
Wild type	c.1504-8T	atcctctgtttttgctgtagGTT	MAXENT: 10.78	MM: 10.84	WMM: 12.46
Mutation	c.1216-6C>A	gcattctgttgtgaacacagGTG	MAXENT: 5.01	MM: 6.48	WMM: 4.10
Wild type	c.1216-6C	gcattctgttgtgaccacagGTG	MAXENT: 7.31	MM: 8.39	WMM: 6.74
Mutation	c.2561-1G>A	caatcatttgccatttctaaGAC	MAXENT: -0.13	MM: -0.56	WMM: -0.35
Wild type	c.2561-1G>A	caatcatttgccatttctagGAC	MAXENT: 8.62	MM: 8.19	WMM: 8.40
Mutation	c.585-1C>T	tctgcttttcattttattaaGCA	MAXENT: -0.59	MM: -0.30	WMM: 1.51
Wild type	c.585-1C	tctgcttttcattttattagGCA	MAXENT: 8.16	MM: 8.45	WMM: 10.26
Mutation	c.1603-1G>A	ctgtgtttttttattcccaaGAC	MAXENT: 1.30	MM: 2.27	WMM: 4.34
Wild type	c.1603-1G	ctgtgtttttttattcccagGAC	MAXENT: 10.05	MM: 11.02	WMM: 13.09
Mutation	c.655-1G>A	cttctttttaactttgacaaATA	MAXENT: -0.50	MM: -0.77	WMM: -0.26
Wild type	c.655-1G	cttctttttaactttgacagATA	MAXENT: 8.25	MM: 7.98	WMM: 8.49

Supplementary Table 4: TP53 mutations in BCC tumors				
**Tumor-ID**	**Genomic position (NC_000017.10)**	**Exon / Intron**	**Mutation (NM_000546.5)**	**Amino acid (CCDS11118)**
1	g.7578401	Exon 5	c.529C>T	p.P177S
4	g.7577058	Exon 8	c.880G>T	p.E294*
11	g.7578212	Exon 6	c.637C>T	p.R213*
13	g.7578212	Exon 6	c.637C>T	p.R213*
	g.7577547_7577548	Exon 7	c.733_734GG>AA	p.G245N
15	g.7578400_7578401	Exon 5	c.529_530del18	p.P177_C182 del PHHERC
	g.7577556	Exon 7	c.725G>T	p.C242F
18	g.7578395	Exon 5	c.535C>T	p.H179Y
	g.7577559	Exon 7	c.722C>T	p.S241F
22	g.7578395_7578396	Exon 5	c.534_535CC>TT	p.H178H; p.H179Y
30	g.7577124_7577125	Exon 8	c.813 814GG>AA	p.V272M
	g.7576855	Exon 9	c.991C>T	p.Q331*
32	g.7577532_7577533	Exon 7	c.748_749CC>TT	p.P250F
	g.7577082	Exon 8	c.856G>A	p.E286K
33	g.7578395	Exon 5	c.535C>T	p.H179Y
	g.7577046	Exon 8	c.892G>T	p.E298*
46	g.7578263	Exon 6	c.586C>T	p.R196*
51	g.7577106	Exon 8	c.832C>T	p.P278S
54	g.7577539	Exon 7	c.742C>T	p.R248T
	g.7577106	Exon 8	c.832C>T	p.P278S
76	g.7577559	Exon 7	c.722C>T	p.S241G
123	g.7578280	Exon 6	c.569C>T	p.P190L
63	g.7579313	Exon 4	c.374G>A	p.E67N
	g.7578472	Exon 5	c.458C>T	p.P153L
	g.7577079	Exon 8	c.859G>A	p.E287K
64	g.7578402	Exon 5	c.528C>G	p.C176T
	g.7577120	Exon 8	c.818G>A	p.R273H
141	g.7578275	Exon 6	c.574C>T	p.Q192*
66	g.7578399_7578400	Exon 5	c.530_531CC>TT	p.P177L
	g.7577531_7577532	Exon 7	c.749_750CC>TT	p.P250L
	g.7577559	Exon 7	c.722C>T	p.S241F
	g.7579313_7579335	Intron 4	c.375-23delAG	
67	g.7578517	Exon 5	c.413C>T	p.A138V
	g.7578263	Exon 6	c.586C>T	p.R196*
7	g.7578212	Exon 6	c.637C>T	p.R213*
70	g.7578212	Exon 6	c.637C>T	p.R213*
	g.7578263_7578264	Exon 6	c.585_586CC>TT	p.I195I; p.R196*

	g.7577099	Exon 8	c.839G>A	p.R280K
16	g.7577509	Exon 7	c.772G>A	p.E258K
	g.7577099	Exon 8	c.839G>A	p.R280K
19	g.7578269	Exon 6	c.580C>T	p.L194F
	g.7578177_7578178	Exon 6	c.672G>A;c.672+1G>A	p.E224E
34	g.7578398	Exon 5	c.531_532CC>TG	p.H178D
50	g.7578253	Exon 6	c.596G>A	p.G199E
81	g.7578466	Exon 5	c.464C>T	p.T155S
	g.7578253	Exon 6	c.596C>T	p.G199V
	g.7578263	Exon 6	c.586C>T	p.R196*
	g.7577559	Exon 7	c.722C>T	p.S241F
84	g.7578471	Exon 5	c.459delC	p.G154fs*16
	g.7577102	Exon 8	c.836G>A	p.G279E
	g.7577106	Exon 8	c.832C>T	p.P278S
	g.7576897_7576898	Exon 9	c.948_949GG>AA	p. Q317*
85	g.7578400	Exon 5	c.530C>T	p.P177L
	g.7578475_7578476	Exon 5	c.454_455CC>TT	p.P152L
	g.7578175	Intron 6	c.672+2A>T	
90	g.7577099	Exon 8	c.839G>T	p.R280I
91	g.7577574	Exon 7	c.707A>G	p.Y236C
94	g.7578275	Exon 6	c.574C>T	p.Q192*
	g.7577547_7577548	Exon 7	c.733_734GG>TT	p.G245F
95	g.7577539_7577540	Exon 7	c.741_742CC>TT	p.N247N; R248T
98	g.7578524	Exon 5	c.406C>T	p.Q136*
102	g.7578212	Exon 6	c.637C>T	p.R213*
	g.7577559	Exon 7	c.722C>T	p.S241G
104	g.7577547	Exon 7	c.734G>A	p.G245D
105	g.7578400_7578401	Exon 5	c.530_531CC>TT	p.P177L
	g.7577082	Exon 8	c.856G>A	p.E286K
106	g.7578400	Exon 5	c.530C>T	p.P177L
	g.7577137_7577138	Exon 8	c.800_801GG>AA	p.R267Q
111	g.7578212	Exon 6	c.637C>T	p.R213*
119	g.7577509_7577510	Exon 7	c.771_772GG>AA	p.L257L; p.E258K
132	g.7578395	Exon 5	c.535C>T	p.H179Y
	g.7577159	Intron 7	c.783-4 G>A	
134	g.7577538	Exon 7	c.743G>A	p.R248P
139	g.7578400	Exon 5	c.530C>T	p.P177L
	g.7577547_7577548	Exon 7	c.733_734GG>TT	p.G245F
146	g.7577559	Exon 7	c.722C>T	p.S241G
	g.7577094	Exon 8	c.844C>T	p.R282T

147	g.7577537_7577538	Exon 7	c.743_744GG>AA	p.R248Q
148	g.7578181	Exon 6	c.668C>T	p.P223L
	g.7577142_7577143	Exon 8	c.795_796GG>AA	p.L265L; p.G266R
150	g.7578398	Exon 5	c.532C>T	p.H178Y
151	g.7578513	Exon 5	c.417G>C	p.K139N
	g.7578549_7578550	Exon 5	c.380_381CC>TT	p.S127S; p.P128S
153	g.7578524	Exon 5	c.406C>T	p.Q136*
160	g.7578400	Exon 5	c.530C>T	p.P177L
	g.7578212	Exon 6	c.637C>T	p.R213*
165	g.7578212	Exon 6	c.637C>T	p.R213*
	g.7577102	Exon 8	c.836G>A	p.G279E
168	g.7578263	Exon 6	c.586C>T	p.R196*
	g.7577082	Exon 8	c.856G>T	p.E286*
170	g.7578395	Exon 5	c.535C>T	p.H179Y
174	g.7577575	Exon 7	c.706T>C	p.Y236H
175	g.7578400	Exon 5	c.530C>T	p.P177L
	g.7578550	Exon 5	c.380C>T	p.S127F
185	g.7578461	Exon 5	c.469G>T	p.V157F
	g.7577498	Intron 7	c.782+1G>A	
186	g.7576899	Exon 9	c.947C>T	p.P316L
187	g.7577547	Exon 7	c.734G>A	p.G245D
189	g.7578400_7578401	Exon 5	c.530_531CC>TT	p.P177L
190	g.7578550	Exon 5	c.380C>T	p.S127F

Supplementary Table 5: Univariate analysis for the effect of mutations in PTCH1, TP53, TERT promoter and DPH3 promoter on patient characteristics																
	**PTCH1**				**TP53**				**TERT promoter**				**DPH3 promoter**			
	**Wt**	**Mut**	**OR (95% CI)**	**P**	**Wt**	**Mut**	**OR (95% CI)**	**P**	**Wt**	**Mut**	**OR**	**P**	**Wt**	**Mut**	**OR**	**P**
**Age**																
<70	31	58	Reference	0.07	57	32	Reference	0.60	42	47	Reference	0.10	61	28	Reference	0.07
>70	49	53	0.58 (0.32-1.04)		69	33	0.85 (0.47-1.55)		36	66	1.64 (0.92-2.93)		56	45	1.75 (0.97-3.17)	
**Gender**																
Men	50	65	Reference	0.58	71	44	Reference	0.13	49	66	Reference	0.54	69	45	Reference	0.71
Women	30	46	1.18 (0.65-2.13)		55	21	0.61 (0.33-1.15)		29	47	1.20 (0.67-2.18)		48	28	0.89 (0.49-1.63)	
**Phototype**																
3 to 5	48	54	Reference		74	28	Reference		48	54	Reference		66	35	Reference	
1 or 2	27	47	1.55 (0.84-2.86)	0.16	41	33	**2.13 (1.13-4.00)**	**0.02**	25	49	1.74 (0.94-3.24)	0.08	42	32	1.50 (0.81-2.79)	0.55
Not stated	5	10			11	4			5	10			8	7		
**Nevus count**																
< 50	63	80	Reference	0.11	98	45	Reference	0.04	55	88	Reference	0.33	88	54	Reference	0.49
> 50	5	15	2.36 (0.82-6.85)		9	11	**2.66 (1.03-6.87)**		10	10	0.63 (0.24-1.60)		14	6	0.70 (0.25-1.93)	
Not stated	12	16			19	9			13	15			15	13		
**Presence of solar lentigos**																
No	6	13	Reference	0.52	11	8	Reference	0.46	8	11	Reference	0.79	11	8	Reference	0.51
Yes, scarce	28	41	0.67 (0.23-1.99)		49	20	0.56 (0.20-1.60)		30	39	0.95 (0.34-2.64)		40	29	1.00 (0.36-2.79)	
Yes, abundant	36	43	0.55 (0.19-1.60)		50	29	0.80 (0.29-2.21)		30	49	1.19 (0.43-3.29)		52	26	0.69 (0.25-1.92)	
Not stated	10	14			16	8			10	14			14	10		
**History of cutaneous neoplasms**																
No	34	33	Reference	0.09	47	20	Reference	0.40	36	31	Reference	0.04	49	18	Reference	0.03

Yes	27	47	1.79 (0.92-3.52)		47	27	1.35 (0.67-2.73)		27	47	**2.02 (1.03-3.97)**		40	33	**2.25 (1.10-4.57)**	
Not stated	19	31			32	18			15	35			28	22		
**Sun-exposure**																
None	42	54	Reference	0.45	62	34	Reference	0.24	37	59	Reference	0.01	62	33	Reference	0.09
<20y	16	14	0.68 (0.30-1.55)		23	7	0.56 (0.22-1.43)		20	10	**0.31 (0.13-0.74)**		22	8	0.68 (0.27-1.70)	
>20y	14	23	1.28 (0.59-2.78)		21	16	1.39 (0.64-3.01)		11	26	1.48 (0.66-3.35)		18	19	1.98 (0.92-4.29)	
Not stated	8	20			20	8			10	18			15	13		
**Immuno-supression**																
No	66	94	Reference	0.15	105	55	Reference	0.95	64	96	Reference	0.36	98	61	Reference	0.34
Yes	6	3	0.35 (0.09-1.45)		6	3	0.96 (0.23-3.97)		5	4	0.53 (0.14-2.06)		7	2	0.46 (0.09-2.28)	
Not stated	8	14			15	7			9	13			12	10		
**Risk type (based on histology)**																
Low risk	21	54	Reference	0.03	47	28	Reference	0.76	20	55	Reference	0.06	41	33	Reference	0.22
High risk	36	44	**0.48 (0.24-0.93)**		52	28	0.91 (0.47-1.74)		33	47	0.52 (0.26-1.02)		52	28	0.70 (0.35-1.28)	
Not stated	23	13			27	9			25	11			24	12		

Phototype: patients classified into five subgroups based on skin complexion and response to sun-exposure

Nevus count represents number of nevi, less than or greater than 50.

History of cutaneous neoplasms represents the past history of skin cancers

Sun-exposure represents cumulative lifetime exposure

Immunosuppression: yes represents patients treated with immunosuppressive drugs like Cyclosporin, methotrexate

Risk type is based on histology, nodular, superficial, and adenoid tumors were grouped as low risk. Infiltrative, morphoeic, micronodular, and metatypical type were categorized as high risk.

Supplementary Table 6: PCR Conditions and Primer Sequences				
**Name**	**Forward primer**	**Reverse primer**	**Tm**	**Product size (bp)**
TERT promoter: -27 to -286	5'CCCACGTGCGCAGCAGGAC 3'	5'CTCCCAGTGGATTCGCGGGC 3'	60°C	260
DPH3-OXNAD1 promoter	5’CGAAGGGGTAACGCCCCAG 3’	5’GGTCCCAGACGTGACGTAGC 3’	56°C	314
TP53: exon 5/6	5'CTGCCGTCTTCCAGTTG 3’	5'CTGACAACCACCCTTAACC 3’	50°C	504
TP53: exon 7	5'AAGGCGCACTGGCCTCATCTT 3'	5'AGTGGGAGCAGTAAGGAGATT 3'	56°C	307
TP53: exon 8/9	5'CTTACTGCCTCTTGCTTCTC 3'	5'CCACTTGATAAGAGGTCCC 3'	50°C	373
PTCH1: exon 3	5’ TCCTATGGCAGGTAGTCAGATAA3’	5’ TAAACCAGCAGCCTTCTCC3’	57°C	342
PTCH1: exon 4	5'TCCAGGGCAACTTCATTTACTA 3’	5’CCAGTCTCATGACTAGAGTTCA 3’	54°C	410
PTCH1: exon 5/6	5’CATTCTGCTGAAATCCCCTCT 3’	5’TACTTGGCAAAAGCTCTGCT 3’	54°C	571
PTCH1: exon 7	5’TGCTCTCCACCCTTCTGAGAG 3’	5’CTCCTACAAGGTGGATGCAGTG 3’	57°C	335
PTCH1: exon 8	5’GCTAGCGAGGATAACGGTTTAAG 3’	5’GCATGTGACCTGCCTACTAATTC 3’	55°C	296
PTCH1: exon 9	5’TGCATAACCAGCGAGTCTGC 3’	5’GAATTGCAGCCAGTGAGTTGG 3’	57°C	295
PTCH1: exon 10	5’CCTGGATGCACATCGATGTT 3’	5’TGTCGAGGCTTGTGGAAGT 3’	56°C	308
PTCH1: exon 11	5’GTAGCATCCTAGTGGAAAAGGC 3’	5’TGATGGGTGGAGGGAAACATTA 3’	54°C	375
PTCH1: exon 12	5’CTTAGGAACAGAGGAAGCTGTGA 3’	5’CTGCTTCAGGAGCTGTTAGGT 3’	54°C	265
PTCH1: exon 13	5’CAGAGCCTCAAACACAGGCA 3’	5’TCACACAGTCTGTGCTCCAAG 3’	56°C	329
PTCH1: exon 14/15	5’CTCCCATGGAAGATGACCTCA 3’	5’AGGCCCAGAAACAGTCAAGAC 3’	57°C	1582
PTCH1: exon 16	5’CTCCACCTGTTACAACATGCTC 3’	5’CTATGGGAGAACAACCCCTACA 3’	54°C	726
PTCH1: exon 17	5’TGCCTTAGGTCTCCAGAGAGCA 3’	5’GCTAGGACCAGGGTCCTTCT 3’	56°C	318
PTCH1: exon 18	5’GTAGCGTCAACGGATGAAGG 3’	5’GTAATGCTGTGCGAAGCTCAG 3’	54°C	375
PTCH1: exon 19	5’ATGCAAGCTATACCCTCCTCC 3’	5’GAGCAGTCAGTAAGCTTTCATGC 3’	56°C	497
PTCH1: exon 20	5’ACTTGGAGACAAACAGAGCCA 3’	5’ATCTGAACCGAGGACACCTTA 3’	54°C	310
PTCH1: exon 21	5’ACCCGGCCCAATCACAATGATT 3’	5’AGATGTGAGCAGTTCTGAGAGC 3’	57°C	334
PTCH1: exon 22	5’ACTGAAGAACCACCAGCAAGTG 3’	5’TGTTCATTTCTGGCGTTGCCAT 3’	57°C	314
PTCH1: exon 23	5’TGTGTGATGTGCTGCTCAGCA 3’	5’CTTTGAGTGTGGCCAGCAGGTA 3’	59.5°C	412
PTCH1: exon 24	5’GACAAAGCTTGGACACATCAGC 3’	5’CTGAGTCTTTGGTGAAACCCA 3’	56.5°C	812
